# Behind the Silence: Training CHWs to Identify and Respond to Social Isolation

**DOI:** 10.1093/geroni/igaf122.3720

**Published:** 2025-12-31

**Authors:** Daniel Moran

**Affiliations:** Dartmouth Hitchcock Medical Center, Grantham, New Hampshire, United States

## Abstract

Social isolation (SI) is a critical social determinant of health that disproportionately affects older adults, increasing the risk of chronic disease, cognitive decline, and mortality. Community health workers (CHWs) are well-positioned to address SI due to their trusted relationships and community-based roles; however, most lack formal training specific to SI. This quality improvement project implemented and evaluated a structured SI training for CHWs at a rural tertiary medical center, guided by Kirkpatrick’s Four Levels of Learning Model. Six CHWs completed pre-training, post-training, and one- and two-month follow-up surveys assessing self-reported knowledge, confidence, and application of an SI component within the Aging Wellness Pathway. The two-hour, case-based training covered distinguishing SI from loneliness, identifying risk factors, communication strategies, connecting to community resources, and integrating interventions into electronic health record workflows. Results demonstrated knowledge gains of 18–26% and confidence gains of 12–32% across 11 SI-related domains, with improvements largely sustained at follow-up. CHWs reported using the SI component in an average of 55% of older adult interactions at two months, facilitating connections to senior centers, companion care, transportation, and the Dartmouth Aging Resource Center. Reported barriers included patient resistance, cognitive decline, and workflow integration challenges. Findings indicate that structured SI training can enhance CHW capacity to identify and respond to SI among older adults, supporting early intervention and connection to evidence-based resources. Sustained use may require ongoing reinforcement, workflow alignment, and strategies to address patient-level barriers.

